# Supplementary material for: Are carbon emissions trading and green financial instruments synergistic? -Comprehensive quantitative research based on content analysis
Source: PLoS One. 2024 Mar 7;19(3):e0298601. doi: 10.1371/journal.pone.0298601 (PMC10919601; doi:10.1371/journal.pone.0298601)
Supplement: S1 Data — (ZIP) [file pone.0298601.s001.zip › Data/地级市Z_F工作报告.zip!/╜¡╦╒/│ú╓▌╩╨/└┤╫╘╣1⁄2╓┌║┼í╛┬φ┐╦╩2╛▌═°í┐_2009.docx]

各位代表：

　　现在，我代表常州市人民政府，向大会作工作报告，请予审议，并请市政协各位委员和其他列席人员提出意见。

　　一、2008年工作回顾

　　过去的一年，是很不平凡的一年。由于国际金融危机的严重影响，我市经济社会发展遇到了前所未有的困难，经受了历史罕见的挑战和考验。市政府在中共常州市委的正确领导下，在市人大、市政协的监督和支持下，紧紧依靠全市人民，坚定信心，积极应对，创新思路，攻坚克难，较好地完成了市十四届人大一次会议确定的目标任务。

　　（一）经济保持平稳较快发展。地区生产总值达到2202.2亿元，按可比价增长12.4%；完成地方一般预算收入185.2亿元，增长17.2%。高效农业加快发展。新增高效农业种植面积11.29万亩，水稻单产和机械化种植率继续保持全省领先。工业结构不断优化。五大产业完成产值2973.5亿元，增长22.8%，其中装备制造业达到1570亿元，新能源产业实现倍增；规模以上高新技术企业完成产值2330亿元，增长24.4%，占规模以上工业的比重上升至44.8%。现代服务业发展加速。实现服务业增加值836.4亿元，按可比价增长13.7%，占三次产业的比重提高到38%；成交额超50亿元的大市场达到7家，超百亿元的3家；软件、动漫产业销售收入分别达到55亿元、9.6亿元；接待国内外游客2066.5万人次，旅游总收入233.9亿元；金融机构本外币存贷款余额分别达到2873.3亿元、1876.8亿元，分别比年初增长24.6%、16.6%。

　　（二）发展活力持续增强。全社会固定资产投资完成1448.2亿元，增长20.3%。其中工业投资831.5亿元，增长22.1%；高新技术产业投资308.6亿元，增长30.8%；服务业投资606亿元，增长17.4%。进出口总额完成176.3亿美元，增长33.3%。其中出口132.4亿美元，增长34.5%；高新技术和机电产品出口比重分别达到15%、54.5%。社会消费品零售总额达到758.2亿元，增长24.1%。国资民资外资齐头并进。11家央企在常增加投资，总额达到57亿元；新增个体工商户26391户、私营企业7842户，注册资本达1038.4亿元，增长16.9%；实际到账外资上报审核数达到22.56亿美元，增长23%，新增工商登记注册外资超3000万美元项目27个，其中1亿美元左右项目10个，天合光能项目超5亿美元。科技创新如火如荼。新认定省级以上高新技术企业198家，新增省级科技企业孵化器9家，新增企业“一站两中心”56家，引进或共建研发机构83家；引进海外人才338名，其中领军型创业人才83名；科教城入驻研发机构和高科技孵化企业超过100家；专利授权2536件，增长15.2%。

　　（三）城乡面貌发生显著变化。区域交通枢纽地位进一步突出。西绕城高速和泰州长江公路大桥南接线开工建设，完成京沪高铁、沪宁城铁常州段拆迁任务，241、338、340省道常州段建成通车，录安洲港区一期建成开放。高架路一期竣工通车，建成晋陵南路、红梅路等跨老运河通道。新建改建运河路、东方西路、竹林北路延伸等骨架道路，扩建龙游路、县学街等次干道和支路。供水、供气、照明、环卫、污水处理等一批市政工程建成投运，北塘河枢纽等城市防洪工程开工建设。建立了数字城管新模式，13项长效管理实现数字化。完成市区13条道路和城市高架路两侧环境综合整治。城市交通管理上升为全国畅通工程A类一等水平。实施城乡绿化十大工程，新增城乡绿地4223.8公顷，国家园林城市获得授牌。水环境治理成效显著。市区46条主要河道完成三年整治目标，基本达到水清标准；3条入太湖河道水质明显好转。节能减排任务如期完成。规模以上万元工业增加值综合能耗估算下降6.2%，化学需氧量和二氧化硫排放总量分别削减6.72%、8.78%。空气质量优良天数达到324天。新农村建设扎实推进。新建和改建农村公路298公里，疏浚县乡河道92条,完成4676个河塘清淤；建成小康家园示范村10个，364个村达到“三清一绿”，266个村达到“五化三有”。

　　（四）改善民生取得新的成效。人民生活水平不断提高。城镇居民人均可支配收入达21592元，增长13.1%；农民人均纯收入达10171元，增长12.6%；城镇新增就业7.9万人，登记失业率控制在3.23%；养老、医疗、失业三大保险综合覆盖率达到97.5%，启动实施新型农村社会养老保险，全面提高城乡低保标准。卫生惠民工程全面展开。社区卫生门诊实行“六免两减”，基本药品实行零差率销售；城镇职工医保住院个人自付比例降低到40%以下，农村新型合作医疗覆盖率达到100%。住房保障工作力度加大。增加廉租房家庭1621户，提供经济适用房3510套。完成120条背街小巷、49.1万平方米老小区整治和2.7万平方米平屋盖修缮，对老小区近400个单元楼道进行整修。菜市场内部提升14个，原址改造3个，搬迁移建11个。提升改造中心城区公厕131座，新建39座。公交优先继续加强。快速公交一号线建成通车，日均客运量稳定在12万人次左右，二号线开工建设；市区行政村公交通达率接近97%，居民公交出行率由12.5%上升到25%。慈善事业稳步发展。慈善基金规模达到14.34亿元，全年救助10万人次。

　　（五）社会事业协调发展。教育现代化步伐加快。常州高职园区成为全省唯一示范高职教育园区，武进、金坛、溧阳通过省教育现代化验收；中小学省级以上优质学校比例达到53%以上，就读学生比例达到67%以上；外来务工人员子女义务教育普及率100%，79%进入公办学校就读。文化事业繁荣发展。市级纪念馆、博物馆、图书馆等公共文化设施全部免费开放；38个乡镇文化体育工作站、60%的村文化室达到省级标准，农村有线电视“村村通”达86%；工人文化宫等场馆改造提升后向社会开放，大剧院主体工程完工，文化传媒中心开工建设；非物质文化遗产保护得到加强，国家级非遗项目达到9项，省级12项。完成市区医院布局总体规划，二院武进阳湖医院、四院新北医院建设进展顺利，戚区医院和广化医院提升为市级医院。体育事业跨越式发展。奥体中心建成开放，常州籍选手获得奥运会金牌1枚、残奥会金牌2枚，成功举办中国羽毛球大师赛、第十三届市运会，成为全省首批体育强市。精神文明建设稳步推进。获得全国创建文明城市和未成年人思想道德建设工作先进市称号。“平安常州”建设取得新成效。城市应急中心投入运行，建成省级“社会治安安全市”。此外，我们还取得了抗击雪灾的胜利，积极支持四川抗震救灾，累计捐赠款物5.26亿元，对口援建工作有序推进。与此同时，安全生产、双拥人防、人民武装、外事侨务、对台事务、审计统计、海关国检、边防海事、邮电供电、档案地方志、防震减灾、民族宗教、老龄、妇女儿童、人口和计划生育、残疾人事业、关心下一代等工作都取得了新成绩。

　　（六）发展环境进一步优化。政务公开加快推进，符合条件的政府信息全部上网公布。改进和加强了新闻发布、网上沟通制度，畅通了政府和企业、市民双向交流渠道。行政审批、行政执法数据库、行政效能监察三网合一，行政许可审批项目集中度由56.5%提高到71%。开展“服务企业关爱有加，支持企业克难求进”活动，围绕“送、减、控、创”出台了一系列政策措施，取消101项行政事业性收费，每年可为企业减负4.6亿元。大力推进依法行政，自觉接受市人大、政协和各民主党派的监督，530件建议和提案全部办结。城市竞争力不断提升，城市综合实力跃居全国第20位，名列“福布斯中国大陆最佳商业城市”第9位，被评为全国优秀创新城市。

　　各位代表，我们之所以能够战胜各种困难，积极应对世界经济危机的挑战，继续保持经济社会健康稳定的发展势头，是全市人民在中共常州市委的领导下齐心协力、克难求进、团结拼搏、锐意进取的结果，也是同方方面面的关心和支持分不开的。在此，我代表市人民政府，向全市人民，向市人大代表、政协委员、各民主党派、工商联、各人民团体、离退休老同志、无党派及各界人士，向驻常部队指战员、武警官兵和全市公安干警，向部、省驻常单位和在常的中外投资者、科技工作者、全体务工人员，向所有关心、支持常州建设和发展的海内外朋友们，表示衷心的感谢和崇高的敬意！

　　在肯定成绩的同时，我们也清醒地看到工作中存在的问题。由于受宏观经济形势的影响，年初确定的地方一般预算收入增长18%的目标没有完成，缺了0.8个百分点；科技创新能力还不强，科技含量高、附加值高的大项目不多，产业结构优化任重道远；环境保护中还存在一些问题，环境污染引发的社会矛盾时有发生；行政机关的效能还不尽如人意，企业对我们的服务还有些意见。这些问题都有待在今后的工作中加以改进。

　　二、2009年目标任务和重点工作

　　当前的经济形势依然十分严峻，我市今年经济和社会发展将面临更大的压力，但是我国经济平稳较快发展的基本面没有改变。只要我们审时度势、积极应对，创新思路、扎实工作，完全可以变压力为动力、化危机为机遇。

　　2009年，我们将以科学发展观为指导，按照扩内需、调结构、重民生、促发展的总体要求，弘扬“创新、发展、提高”主旋律，把加大投入、扩大内需作为促进经济增长的根本途径，把转变方式、调整结构作为提升发展水平的主攻方向，把科技创新、扩大开放作为经济发展的重要动力，把以人为本、改善民生作为一切工作的出发点和落脚点，努力实现经济社会又好又快发展。

　　全市主要预期目标是：地区生产总值增长10%；地方一般预算收入增长10%；全社会固定资产投资增长17%，其中工业投资增长20%；社会消费品零售总额增长18%；注册外资实际到账增长10%；全社会研究与开发经费支出占地区生产总值的比重达到1.9%；城镇居民人均可支配收入增长10%，农民人均纯收入增长10%；城镇登记失业率控制在4%左右；万元地区生产总值综合能耗下降4.5%，化学需氧量和二氧化硫排放量均削减2%；环境质量综合指数达到83分以上。

　　为实现上述目标，今年将着力抓好以下七项重点工作：

　　（一）采取切实措施，确保经济增长

　　今年可能是新世纪以来经济发展最困难的一年。制止经济下落，确保平稳增长，是今年工作的重中之重。

　　1．加大投资保增长。（1）加快重大产业投入。重点实施高效农业、先进制造业、现代服务业项目。（2）加快重大基础设施投入。重点实施铁路、港口、航道、机场、高速公路和水利能源等项目。（3）加快重大城乡环境投入。重点实施城乡绿化、污水垃圾处理、水环境治理、节能减排等项目。（4）加快重大社会事业投入。重点实施教育、文化、体育、卫生等项目。（5）加快重大民生工程投入。重点实施就业、社会保障、公用事业等项目。全面实施50项重点工程，推进169个市级重点项目，完成全社会固定资产投资1700亿元以上。

　　2．刺激消费保增长。（1）提高居民消费能力。更加关注中低收入居民，提高城镇居民养老金发放标准，提高城乡居民最低生活保障标准，提高优抚对象等人员抚恤和生活补贴标准，适时增加对生活困难群众和家庭经济困难学生补助。（2）着力培育消费热点。积极开发旅游、文化、健身和网络等热点消费；大力发展社区商业、物业、家政等服务性消费；通过家电下乡、建设农村服务体系等举措，大力开拓农村消费市场。（3）促进居民住房消费。落实更加积极的政策措施，激活房地产市场，力争全年销售商品房400万平方米以上。

　　3．强化“三农”保增长。（1）千方百计增加农民收入。确保农村经济总收入增长20%以上，培训农村劳动力2.5万人；落实粮食补贴、失地保障、农村低保等各项惠农政策，着力提高农民收入水平。（2）深化农村改革。探索组建镇级股份合作联社，新增农民专业合作社60家、土地股份合作社20家；扩大农业保险覆盖面，抓好集体建设用地使用权流转试点，推进农村土地承包经营权流转，新增流转面积10万亩。（3）实施农业“双百万亩”工程。高标准优质粮田五年内达到100万亩，今年完成20万亩；高效农业面积三年达到100万亩，今年新增10万亩。

　　4．扩大开放保增长。（1）稳定发展外经外贸。外贸出口增长10%以上，高新技术产品、机电产品比重都提高3个百分点；对外承包工程营业额和中方境外投资额均增长10%。（2）突破外资大项目。新增总投资超亿美元或工商登记注册外资超3000万美元项目15个，力争超3亿美元的2个，超5亿美元项目取得新突破，世界500强企业投资项目5个。（3）实施园区建设跃升工程。园区基础设施投入100亿元以上，实际到账外资、国内生产总值、一般预算收入占全市比重分别超过70%、40%、45%，外资大项目占全市比重达到70%以上。

　　5．激活企业保增长。（1）提振信心。引导企业正确认识国际国内形势，抢抓国际经济大变化、大调整的机遇，迎难而进，发展自己。（2）扩大市场。支持企业加快调整产品结构，适应消费和市场需求，加大国际市场开拓力度，做到国内、国际两个市场一起抓。（3）融通资金。全面贯彻适度宽松的货币政策，为企业发展创造良好的融资环境，支持企业通过信贷、上市等形式募集更多的发展资金。今年信贷规模总量、中小企业贷款均增长15%以上。

　　（二）加快结构调整，培育优势产业

　　加快产业结构调整，是解决经济深层次矛盾、有效应对外部冲击的根本出路，也是远近结合、造就我市产业优势的战略需要。

　　1．全力振兴五大产业。（1）制定实施振兴五大产业三年行动计划。到2011年，产业销售规模实现翻番，达到6000亿元以上；今年要超过3000亿元，占规模以上工业比重提高1个百分点。（2）实施“千亿产业、百亿企业”培育工程。通过三年努力，把输变电设备、新能源、新材料打造成千亿级产业，其中新材料产业今年力争销售超千亿；着力发挥大企业对五大产业的支撑作用，三年新增年销售收入超百亿的企业4家以上，今年力争1家。（3）抓好重点项目、重点企业。实施50个五大产业重点项目，今年新开工13个，竣工17个；重点扶持行业龙头企业，促进做强做大。（4）推进企业联合重组。抓住当前产业整合的有利契机，支持企业走兼并、重组、联合的道路。

　　2．加快发展现代服务业。（1）建设十大重点市场。年成交额超50亿元的大市场达到10个以上，超100亿元的达到3个。（2）推进物流业与制造业联动发展。全面提升第三方物流水平，中国物流公司常州分公司、常州城西物流园区配送额分别达到30亿元和20亿元。（3）推进旅游业重点项目建设。编制休闲旅游发展规划，重点建设缤纷亚洲、南山竹海温泉等十大休闲旅游项目。全年接待国内外游客2200万人次，实现旅游总收入250亿元。（4）大力发展新型商业业态。重点打造武进花园街、天宁九洲新世界、钟楼五星等三个新型商业街区。（5）加快创意产业发展。整合提升公共技术服务平台，建设服务外包人才培训基地，加快发展以软件、动漫为主体的创意产业。新增创意企业80家，产业销售达到60亿元。（6）加强金融服务创新。实现企业境内外上市各1家，申报5家。确保2家村镇银行、4家小额贷款公司开业。引进3家外地金融机构来常开设分支机构。

　　3．突出科技创新。（1）加快发展高新技术产业。规模以上高新技术产业产值达到2800亿元，其中五大产业占90%以上；新增省级以上科技项目160项，通过国家新标准认定的高新技术企业70家以上；重点扶持100家创新型试点企业，企业研发投入达到90亿元。（2）加快建设科技创新三级平台。新增企业“一站两中心”50家、市级以上科技公共服务平台15家，引进和培育省级外资研发机构10家；孵化器孵化面积累计达200万平方米，在孵企业累计2000家；加快科教城三期国际创新基地建设，引进研发机构及高科技企业80家。（3）深化产学研合作。积极开展先进制造技术成果洽谈会等对接活动，组织3000家民营企业参与产学研合作，新增重大合作项目100项以上。

　　4．锲而不舍引进人才。全年引进各类人才25000人以上，其中硕士以上紧缺高层次人才1200人；引进40名领军型海归创新创业人才、60名高层次研发人才，力争20人入选省高层次创新创业人才，全年引进海外人才总数超过400名，签约项目落户率超过70%。

　　5．加大对重点产业支持力度。（1）设立专项基金。市财政预算外每年安排3亿元、辖市区配套3亿元，建立五大产业专项扶持基金，重点支持企业科研开发、技术装备升级、平台建设、人才引进和资本运作。（2）集中政策资源。梳理归并现有产业扶持政策，变部门分散管理为全市统筹管理，变面上扶持为重点扶持，集中投向五大产业和现代服务业重点企业和重点项目。（3）积极向上争取。加强与国家、省产业政策对接，争取更多项目列入国家和省重点项目，争取获得更多的上级科技和资金支持。

　　（三）抢抓政策机遇，加快基础建设

　　抓住扩大投资机遇，实施一批事关我市全局和长远的重点基础设施工程，不仅对促进经济回升起到积极的作用，而且可以为今后发展打下坚实的基础。

　　1．加快交通枢纽建设。（1）“三铁六站”建设工程。完成沪宁城际铁路、京沪高速铁路土建及架梁工程，开工建设宁杭铁路客运专线；沪宁城际铁路常州站、戚墅堰站主体工程完工，京沪高铁常州站开工建设，实施戚墅堰车站货场搬迁，宁杭铁路客运专线溧阳站、瓦屋山站完成前期工作。（2）机场改扩建工程。年内完成飞行区跑道改造，完成航站楼及配套设施建设工作量的50%，明年年底前建成开放。（3）高速公路建设工程。西绕城高速和泰州长江公路大桥南接线完成软基处理、涵洞、桥梁桩基建设。（4）干线公路建设工程。完成通江大道、长虹路、录安洲港区疏港道路改造，实施104国道、241省道部分路段一级路改造。（5）录安洲港码头建设工程。建成2个万吨级石化码头、6个夹江千吨级泊位。（6）三级航道网整治工程。加快推进锡溧漕河、京杭运河西段整治工程。

　　2．推进“三城联动”。（1）做好重点区域规划。完成城铁站核心区周边地区规划、高铁站周边地区综合交通规划和城市设计，编制奔牛机场周边空、铁、公、水交通枢纽地区规划。（2）实施中吴大道城市化改造。对中吴大道玉龙路至天宁大桥段进行城市化改造，年内快车道建成通车。（3）加快武进新城区建设。新建续建延政东路延伸段等五条道路，对长虹中路进行黑色化改造，加速推进滆湖保护利用一期工程，继续实施太湖清淤、漕桥河整治等工程。（4）推进新北新城区建设。完成高铁站征地拆迁及辽河路、常新路等场站周边主要道路建设，飞龙西路等五条道路建成通车。全面实施122省道、省庄河两侧等绿化工程。（5）继续推进东大门建设。启动建设沪宁城际戚墅堰站广场，开工建设五一路南段，完成戚机厂老工房区和常丰河整治任务。

　　3．加快城市基础设施建设。（1）实施城际铁路配套工程。同步建设新堂北路、竹林西路，开工建设飞龙东路、永宁路及地道、戚月路及地道、竹林南路及地道、泡桐路及地道，以及大明路、玉龙路等过铁路通道。（2）加密城市道路网。拓宽清潭路、吊桥路、月季路等次干道与支路。（3）继续实施城市防洪大包围工程。开工建设大运河东枢纽、串新河枢纽、南运河枢纽。启动新沟河拓浚延伸工程。

　　（四）优化人居环境，建设生态城市

　　节约能源资源、保护和建设生态环境，功在当代，利在千秋，决不能因为经济遇到困难而有丝毫放松。

　　1．绿化美化环境。（1）大面积绿化城乡。实施生态修复、绿色通道、河道生态绿化、城乡道路绿化提升、城市出入口绿化、城市公园增绿、镇村绿化、城市公共服务空间增绿等八大绿化工程，新增绿地1663.94公顷。（2）建成“三河三园”景观河带。按照“现代、亲水、生态、文化”的要求，全面完成关河、北塘河、东支河河道及两侧地区综合整治，贯通东坡公园、红梅公园、恐龙园，使之成为城市建设和旅游的新亮点。

　　2．巩固长效管理。（1）提升数字化城市管理。数字化城管覆盖面积扩大到220平方公里；构建全方位视频监控系统，对管理区域实行动态实时监控；建立市、区、街道城市管理三级平台。（2）继续实施市容环境综合整治。对清潭路、健身路、荆川路等省运会比赛场馆主要出入口道路，快速公交2号线、长虹东路、惠山南路等23条道路两侧进行市容环境综合整治；对延陵西路、勤业路等已整治道路进行提升和完善。

　　3．提升环境质量。（1）进一步改善水环境。继续开展市区河道整治，逐步达到水清岸绿景美要求；3条入太湖河道水质全年稳定达到五类标准，总氮、总磷排放量下降5%。（2）提高空气质量。对工业废气、施工扬尘、机动车尾气、秸秆焚烧、燃煤等废气污染开展专项整治，关闭10家重点污染源企业，搬迁常隆化工、光辉化工、华达化工、天马集团等10家企业，整治100家排放不达标企业。（3）推进节能减排。完成82家重点企业和8家工业污水处理厂、6家城市生活污水厂的提标改造，对37个乡镇实施污水集中处理，建成投运100个农村生活污水处理工程，电力企业全部实施脱硫运行，建设工业固体废弃物安全填埋场一期工程。（4）全面提升城市污水处理能力。江边污水厂二期、武南污水厂、金坛第二污水厂二期和溧阳第二污水厂建成投运，新增日污水处理能力23.5万吨；铺设污水主管道30公里，建成泵站6座。

　　（五）统筹协调发展，繁荣社会事业

　　加大社会事业建设力度，既是扩大内需、促进经济增长的重要举措，也是统筹发展、建设和谐社会的内在要求。

　　1．全面推进教育现代化。（1）所有辖市区都要达到省教育现代化标准。（2）刘国钧高等职业学校新校区完成主体工程建设。（3）旅游商贸高等职业学校全面竣工。（4）西藏民族中学实施整体搬迁，年底建成投用。（5）蓝天实验学校实施整体搬迁改造，完成一期工程。（6）开工建设新北实验初中改扩建工程。

　　2．加强卫生基础设施建设。（1）新建改建城市社区卫生服务中心8个、服务站23个。（2）年内建成市中医院门急诊病房楼、三院门急诊病房楼、疾病预防控制中心综合楼。（3）开工建设市儿童医院病房楼，启动建设中医院钟楼院区，实施第七人民医院改扩建工程。（4）完成阳湖医院主体工程、新北人民医院主体土建工程。（5）完成数字卫生信息系统工程一期建设。

　　3．繁荣发展文体事业。（1）常州大剧院建成开放。（2）开工建设少年科学艺术宫三期、亚细亚影视城综合功能开发提升工程。（3）加快推进现代传媒中心建设。（4）完成刘海粟美术馆改造提升。（5）实施盛宣怀故居修缮工程。（6）全面整修十七届省运会体育场馆，完成16个比赛场馆维修整新、清潭体育馆和体育场大修工程，奥体中心综合服务大楼年底前竣工投用。

　　4．协调发展其他各项事业。深入开展“双拥模范城”创建活动，不断提升双拥工作水平。继续加强人口和计划生育管理服务，支持发展妇女儿童、老龄和残疾人事业，全面做好外事、侨务、台湾事务、地方志、民族宗教、关心下一代等工作。

　　（六）坚持以人为本，着力改善民生

　　越是经济困难，越要关注民生，保障和改善民生，切实解决事关人民群众切身利益的热点和难点问题。

　　1．不断提高人民收入水平。（1）努力扩大就业。加大财政投入力度，扩大失业保险基金使用范围，加强就业培训，积极开发公益岗位，全市充分就业社区创建率达80%以上。（2）支持创新创业。降低准入门槛，大力发展个体私营经济，多渠道增加人民群众财产性收入、经营性收入。新增个体工商户1.5万户、私营企业6000户、注册资本80亿元。（3）完善提高社会保障。养老、医疗、失业保险分别净增3万、3万、2万人，三大保险综合覆盖率保持在97%以上；继续提高老保、医保、低保和慈善救助水平。

　　2．人民共享发展成果。（1）加强公共卫生服务。完善公共卫生体系，提高饮用水水质检测标准，增加儿童免费预防接种项目，加强农民免费公共卫生服务、妇女病普查及重大传染病防控工作。（2）提升基础教育水平。全市早期教育普及率达90%以上，省级优质幼儿园占比达到60%，就读人数达70%以上；省级优质中小学占比达到60%，就读人数达70%以上；外来务工人员子女接受义务教育普及率100%、公办学校吸纳比例80%以上；新增各类特色学校80所。（3）丰富群众文体生活。行政村文化室省标达标率超过90%，完成所有行政村农家书屋建设布点，农村有线电视“村村通”达92%；开展阵地演出、广场演出、社区流动演出等多种群众喜闻乐见的文化活动；完善奥体中心全民健身设施建设，对现有170个全民健身点进行整新，100%乡镇健身中心达省标，新建小区配套体育设施100%达标。（4）改革经济适用房补贴办法。变暗补为明补，把补“砖头”改为补“人头”，实行货币化补贴。同时，加大廉租房和公共租屋建设力度，到明年底全面实现“小康常州居者有其屋”的目标。

　　3．大力发展公用事业。（1）加快公共交通设施建设。建设钟楼、新龙、城铁常州站、城铁戚墅堰站等公交枢纽和一批公交首末站、停靠站；建成公交调度指挥中心和公交智能化系统，提升公交运营调度的科学管理水平。（2）提高城市供水能力。续建魏村水厂，新增日供水能力30万立方米，中心城区和新北区日供水能力突破100万立方米。（3）继续改造提升菜市场。建设盘龙苑、九龙等10个菜市场，推进新建小区配套菜市场建设。（4）完成公共厕所改造提升工程。新建公厕32座、改造公厕75座。

　　4．为民办好十件实事。（1）扩大就业。新增就业岗位8万个，城镇新增就业6万人，农村劳动力转移1万人，援助困难群体就业3000人，动态消除城镇零就业和农村零转移家庭。（2）建立新型农村社会养老保险制度，提升市区社会养老服务水平。金坛、溧阳、武进、新北新型农村社会养老保险覆盖面达到60%以上；全面实施农村居民养老补贴制度。规划建设市区老年人日托站和社区老年助餐服务点。市区居家养老服务补贴标准由每月100元提高到200元。（3）加快住房保障步伐。筹集住房保障资金30亿元，年内全部解决符合经济适用房申购条件的购房者补贴，每户补贴8万元；新增廉租房、公共租屋1500户以上；住房公积金扩面5万人以上，发放低息贷款13亿元；对65万平方米老住宅小区进行综合整治，2.9万平方米房改房进行平屋盖修缮。（4）方便群众就医，减轻医疗负担。社区卫生服务机构免费为居民提供公共卫生服务，按成本收费提供基本医疗服务，零差率药品增加品规至300种以上，门诊平均费用比医院低50%以上；新型农村合作医疗全市人均筹资不低于190元，金坛、溧阳180元，住院费用实际补偿比例达到40%；在常高校学生纳入城镇居民医保，城镇职工医保人均住院自付比例降到35%；全市范围实行免费婚检和孕前优生检测。（5）改善农村办学条件。实施农村中小学千万元以上教育项目16个，新建校舍30万平方米以上；全市独立建制公办中小学办学条件达省二类标准以上。（6）继续实施公交优先。快速公交2号线建成通车；新增空调车400辆，市区空调车比例达60%以上；新辟公交线路15条，调整优化18条；规范出租车管理，60%的出租车错开交接班时间。（7）新增公园绿地。改造提升东坡公园，建成圩墩公园、西林公园、华山公园、飞龙公园，并向市民免费开放；建成恐龙谷温泉公园，建设紫荆公园，新建武进聚湖公园，启动建设丁塘河湿地公园一期；新增公园绿地64.9公顷。（8）建设小康家园。建成10个小康家园示范村，300个行政村达到“三清一绿”，100个达到“五化三有”；新建农村公路140公里，改造农村危桥100座,疏浚县乡河道90条，整治村庄河塘4000个。（9）实施蓝天碧水工程。稳定提升市区已整治46条主要河道水质，新整治14条支线河道，年内6条达到水清标准；对城区120平方公里内废气排放进行全面整治，扩大高污染燃料禁燃区8.1平方公里，城市环境空气质量优良天数达330天以上。（10）建设平安小区。市区建立流动人口服务管理中心98个，建成技防社区、村156个，补建49个住宅小区技防设施，对发案较多的散居居民楼安装楼宇电子防盗门，在老住宅小区及周边增设停车泊位5000个。刑事案件发案率小于万分之七十二，公众安全感达到95%以上。

　　（七）加快转变职能，强化自身建设

　　在当前大变化、大调整中，更要加快政府职能转换，加强自身建设，不断优化发展环境。

　　1．服务企业发展。（1）让企业轻装上阵。认真落实国家、省市各种优惠政策，清理不规范收费和罚款，切实减轻企业负担。（2）为企业雪中送炭。想企业所想、急企业所急、解企业所难，为企业提供急需的金融、工商、税务、用工等各种服务。（3）让企业专心致志。坚持“有求必应，无事不扰”，严格执行宁静生产日制度，控制和减少检查。（4）助企业创新创造。企业在没有违反国家法律规定的前提下的一切试验和探索，都要以满腔热情的态度予以鼓励和支持，为他们提供所需要的服务。

　　2．推进政务公开。（1）确保权力阳光操作。完善重大问题专家咨询、公示听证、集体决策制度，提高决策透明度和公众参与度，保障市民的知情权、参与权、表达权和监督权。（2）提升电子政务。加快建设信息资源共享、关联业务互动、服务一站式的电子政务网络体系，完善“三合一”网络平台，形成省、市、县三级联网体系。（3）畅通民意渠道。完善新闻发布制度，办好市长信箱、市长与网民等交流平台，广泛吸纳民智民慧，及时发布重要信息。（4）提高审批效率。加强项目审批集中管理，即办件占比提升至70%，承诺时限在法定时限的基础上压缩40%以上。

　　3．严格依法行政。（1）贯彻国务院《关于加强市县政府依法行政的决定》，严格按照法定权限和法定程序行使权力、履行职责。（2）落实行政执法责任制，做到严格执法、规范执法、文明执法。（3）自觉接受人民代表大会及其常务委员会、人民政协的监督，接受司法、新闻舆论和社会公众的监督，认真听取方方面面意见，不断改进政府工作。

　　4．坚持勤俭廉政。（1）优化支出结构。节约政府开支，市级机关公用经费在去年基础上零增长，将财力更多地投向创新创业和社会民生事业。（2）开展专项整治。重点解决好生态环境保护、食品药品质量、安全生产、征地拆迁、农村集体资金使用中群众反映强烈的问题，维护社会和谐稳定。（3）坚决反腐倡廉。全体公务员要严格要求自己，处处廉洁自律；进一步完善惩治和预防腐败体系，严肃查处违纪违法案件，惩处腐败分子。

　　各位代表，虽然前进道路不平坦，但发展前景很光明。让我们在中共常州市委的正确领导下，以披荆斩棘的勇气、创新创造的举措、务实高效的作风，顽强拼搏，克难求进，为夺取今年经济社会发展的新胜利而努力奋斗！
